# Differentiation of self and relationship attachment, quality, and stability: A path analysis of dyadic and longitudinal data from Spanish and U.S. couples

**DOI:** 10.1371/journal.pone.0282482

**Published:** 2023-03-02

**Authors:** Martiño Rodríguez-González, Chance A. Bell, Sergio B. Pereyra, María Pilar Martínez-Díaz, Maria Schweer-Collins, Roy A. Bean

**Affiliations:** 1 Institute for Culture and Society (ICS), Universidad de Navarra, Navarra, Spain; 2 Department of Counseling, School Psychology, and Family Science, University of Nebraska Kearney, Kearney, NE, United States of America; 3 Department of Counselor Education and Rehabilitation, California State University, Sacramento, CA, United States of America; 4 Psychology Department, Faculty of Humanities and Social Sciences Universidad Pontificia Comillas, Madrid, Spain; 5 Prevention Science Institute, University of Oregon, Eugene, OR, United States of America; 6 School of Family Life, Brigham Young University, Provo, UT, United States of America; The Ohio State University, UNITED STATES

## Abstract

**Objectives:**

In the current study, we examined the relationship between differentiation of self (DoS) and key relationship functioning variables among couples. This is the first study to test such relationships using a cross-cultural longitudinal approach (i.e., samples from Spain and the U.S.) while controlling for stressful life events–a key theoretical construct in Bowen Family Systems Theory.

**Methods:**

A sample of 958 individuals (*n* = 137 couples from Spain, and *n* = 342 couples from U.S.) was used in cross sectional and longitudinal models to analyze the effects of a shared reality construct of DoS on anxious attachment, avoidant attachment, relationship stability, and relationship quality while considering gender and culture.

**Results:**

Our cross-sectional results indicated that men and women from both cultures experienced an increase in DoS over time. DoS predicted increased relationship quality and stability and decreased anxious and avoidant attachment in U.S. participants. Longitudinally, DoS predicted increased relationship quality and decreased anxious attachment for Spanish women and men, while it predicted greater relationship quality and stability and decreased anxious and avoidant attachment of U.S. couples. Implications of these mixed findings are discussed.

**Conclusions:**

Higher levels of DoS are linked with a better couple relationship across time, despite varying levels of stressful life events. Although some cultural differences regarding the links between relationship stability and avoidant attachment exist, this positive link between differentiation and the couple relationship is mostly consistent across the U.S. and Spain. The implications and relevance for integration into research and practice are discussed.

## Introduction

Understanding relationship functioning and how it can be improved, whether through prevention, training, or psychotherapeutic interventions/programs, is a high value research goal [1–4]. This is particularly important given that healthy and happy relational functioning is a strong predictor of psychological and physical health, and better social and work functioning [5, 6]. Among the variables and theories developed to understand relationship functioning, differentiation of self (DoS), the cornerstone construct of Bowen Family Systems Theory (BFST), has received increasing empirical support [7, 8]. Differentiation can be conceptualized as both: (a) an individual’s awareness of the differences between emotional and intellectual processing, and (b) a dyad’s ability to engage in a healthy balance of connectedness and separateness. Given its association with individuals’ capacity to self-regulate within the context of their significant relationships, it is especially relevant to couples therapy and relational psychoeducation programs [2, 9].

Notwithstanding increased research attention to DoS, there are several key limitations associated with this growing body of research that are directly countered in this study. First, the majority of studies of DoS (a relational variable) have maintained an individual perspective or have utilized an actor-partner interdependence (APIM) model [3, 10–12]. The APIM approach is one possible way to statistically analyze the relationship as a system among others [13]. In the current study, we created a DoS shared reality variable. A shared reality perspective is another way to statistically deal with the complexities that data from multiple system members present, allowing for the combination of individual scores to create a sense of shared meaning [13]. We chose an analytical approach informed by Busby and colleagues’ shared reality method. We posited that combining the individual self-reported perceptions of DoS from each member of the dyad would result in a sense of the emotional climate of the couple system. This aligns with Bowen’s theory that the family functions as an emotional unit [9, 14]. The use of a path analytical approach permitted us to examine how a single predictor (combined DoS scores) would influence multiple relational variables simultaneously. Doing so provided a clearer picture of the influence the emotional climate of the couples’ relationship had on these relational variables. Second, very few studies have examined DoS in relation to key relational variables (e.g., anxious attachment, avoidant attachment). Finally, relatively few DoS studies include an examination of longitudinal data [10].

Addressing these limitations, the current study examines DoS (at time 1) across time with other relational outcomes (at time 2), including relationship quality and stability and anxious and avoidant attachment. In an effort to further examine the context in which DoS and couple outcomes occur, we controlled for the effect of stressful life events. Moreover, we explored these relationships using a culturally diverse sample of couples from two countries (i.e., Spain and the U.S.), with longitudinal and dyadic data (from both members of the couple). Although Bowen´s theory claims that DoS is universal and thus applies to all cultures [14], there has been some research pointing to the idea that it is modulated by the cultural background [8]. Therefore, considering cultural and gender factors will aid researchers in the development of personalized prevention programs and/or couples therapy.

## Literature review

### Differentiation and attachment in relationship functioning

#### Differentiation and attachment conceptualizations

Bowen family systems theory (BFST) provides an understanding of how early family relations shape the development of adult couple bonds [7]. Bowen found that under stressful situations, individuals high in DoS were more successful in balancing thinking and feeling systems as individuals [13], and autonomy and intimacy needs as individuals/couples. Conversely, individuals with low levels of DoS were more likely to display behavior predominately governed by automatic emotional responses, such as emotional isolation, emotional flooding, or lability [15].

Relatedly, attachment helps us understand our responses in intimate relations based on two dimensions: anxiety and avoidance [16]. Avoidant attachment relates to deactivation of attachment and emotion-regulation strategies (e.g., suppression of attachment related thoughts and emotions). Anxious attachment relates to hyperactivation of attachment and emotion-regulation strategies (e.g., insistent attempts to obtain care, support, and love from their partner). Attachment security, characterized by comfort with closeness and trust in the availability, responsiveness, and supportiveness of their partner, is related to low levels of anxiety and avoidance.

#### Relationship of differentiation and attachment

Researchers have suggested ways BFST and attachment could explain intimacy and closeness in couple and other interpersonal relationships in a complementary way [7, 17, 18]. More specifically, individuals low in DoS often employ two common strategies to regulate anxiety: emotional cutoff and emotional reactivity, which correspond to avoidant and anxious responses, respectively, which are theoretically associated with insecure attachment styles. Avoidant individuals, when under stress, tend to suppress emotional connections and needs and avoid closeness, behaviors akin to emotional cutoff. Anxious individuals are preoccupied with abandonment, demand a supportive and caring response when they feel threatened or distressed, and protest perceived unavailability or lack of responsiveness–similar to, yet distinct from, emotional reactivity [19]. Couples research supports an empirical connection among maladaptive affect regulation and anxious and avoidant attachment behaviors [17, 20–22]. More specifically, three studies reported an association between emotional cutoff and avoidant tendencies, and between emotional reactivity and anxious tendencies [17, 18, 21].

Although interest continues to grow regarding the link between differentiation and attachment in couples therapy [7], a paucity of empirical research focusing on relationship variables with couples persists. Based on this review of the available literature, only three studies focusing on couple relationships linked DoS and attachment [17, 22, 23]. These studies found both differentiation and attachment variables relevant to relationship outcomes (e.g., dyadic adjustment). First, Dell’Isola and colleagues assessed anxious attachment in a sample of North American university students and found lower levels of DoS resulted in higher anxious attachment levels seven weeks later [23]. Second, Timm and Keiley found [22], in a sample of 205 married adults living in the midwestern U.S., that higher differentiation was significantly (positively) associated with secure attachment, and their model suggested attachment could have a direct impact on relationship adjustment. They also found DoS had a positive impact on relationship adjustment, but this effect was indirect and mediated through sexual communication. Third, Lampis and Cataudella conducted a study with 350 romantically involved Italian adults [17]. Their results also showed higher DoS was associated with more secure attachment. However, they did not include measures of relationship adjustment or satisfaction.

In sum, extant research supports DoS and attachment as potentially interrelated constructs with complex interlocks, requiring further research. Nevertheless, the aforementioned studies share a common limitation in that they did not study both partners [17, 22, 23], thereby limiting a clearer understanding of how the two constructs operate within couples and how interventions might interrupt maladaptive relational patterns. As a clear strength, we designed our study to explore both DoS and attachment variables with a sample comprised of both partners in each couple.

### Differentiation of self and relationship outcomes: Filling the gaps

The positive impact higher differentiation levels have on relationship quality has support from several empirical studies in the last two decades [8, 24]. Empirical research validates Bowen’s hypothesis that individuals who report higher DoS levels have better relationship quality as they can experience emotional intimacy without renouncing their sense of autonomy.

#### Differentiation of self and relationship stability

Most previous studies that tested how DoS influences relationship functioning focused on dyadic adjustment or relationship satisfaction. In our study, we built upon their work, seeking to replicate their findings by examining the relationship between DoS and relationship quality [25]. Simultaneously, we focused on extending this examination of potentially co-occurring interactions by analyzing relationship stability. While some researchers have conceptualized (and assessed) relationship stability as a component of relationship quality [11], other convincing evidence suggests that the stability of and satisfaction in a relationship represent two distinct constructs [26, 27]. More specifically, an individual may remain in a relationship (i.e., stability) and not find satisfaction in it and, on the other hand, satisfaction may be relatively high, yet the individual remains open to finding a “better” partner. Relationship stability, directly assessed, has received little attention in connection with DoS, with one exception. Cabrera García and colleagues [28], with a sample of Colombians, found that DoS did not correlate significantly with relationship stability.

#### Differentiation of self and longitudinal data

The limited number of studies utilizing longitudinal data represents another relevant gap in the DoS literature and relationship variables. To our knowledge, only two studies analyzed DoS and relationship outcomes with longitudinal data, both with U.S. couples. First, Bartle-Haring et al. found that DoS mediated the relationship between depression and relationship satisfaction among 412 North American couples [10]. Second, Dell’Isola and colleagues found differentiation did not significantly predict relationship satisfaction directly or indirectly among 162 romantically involved university students from the U.S. [23].

### Differentiation of self and stress

The scarcity of longitudinal data collected from both relationship partners led to another significant limitation. When reviewing studies that explore the association between differentiation and relationship functioning, the majority did not control for stress, which we defined as an occurrence of significant magnitude to potentially provoke anxiety and change in a family system. We found no empirical studies that examined the impact of differentiation on relationship functioning (e.g., relationship adjustment, relationship quality, relationship stability, etc.), and which controlled for the effects of stressful events. Stress, and specifically stressful life events, are a key variable in Bowen’s theory, with Kerr and Bowen positing that “people at any point on the scale (of differentiation), if stressed sufficiently, can develop physical, emotional, or social symptoms.” [29, p.97]. This implies that a couple with lower levels of differentiation could exhibit a high degree of relationship adjustment in low stress circumstances. In addition, Bowen posited that the couple functions as an emotional system, so it is necessary to control for the stressful life events each partner endures.

Prior research on couples shows the relevance of stress to relational functioning [5, 6, 30, 31]. Couples can face numerous stressful events and situations over a lifetime. The ability to cope with stress and make a healthy adjustment is key for maintaining relationship satisfaction [1]. Furthermore, stressful life events and their accumulation increase the degree of pressure on couples and difficulty when dealing with other stressors [32]. According to Bowen, DoS is the key factor for dealing with stress and reducing relationship distress by minimizing its negative impact on the individual and the relationship. Considering that couple stress models emphasize the dyad’s capacity for coping with stress [1], exploring the relationship between the couple’s joint DoS and relationship outcomes, while controlling for stressful life events, will help us understand the role DoS plays in relationship functioning.

### Current study: Strengths, goal, and research questions

This study is the first, to our knowledge, to (a) use a cross-cultural longitudinal approach (i.e., samples from Spain and the U.S.), (b) assess both differentiation and attachment variables in a Spanish sample of couples, and (c) use a “shared reality” framework, with the DoS variable, to validate the hypotheses in question.

Bowen’s theory and previous studies suggest that similar patterns exist across cultures and gender [8]; however, prior research also indicates that some cultural and gender differences exist in the relationship between DoS and relational outcomes, for example in the relevance of emotional cutoff [22, 33, 34]. This study contributes to an understanding of the impact of DoS on relationship functioning in areas of attachment and relationship quality and stability. It also contributes to the advancement of DoS because we considered the influence individual level variables had on couple level variables from a novel perspective in the DoS literature. Using a shared reality approach and exploring our research questions with two different cultures, we examined the reality of the couple as an emotional unit, as postulated by Bowen [14]. To that end, in accordance with Bowen’s theory [14], we considered DoS as a shared reality variable.

Thus, the goal of this study was to add clarity to the relationship between DoS and the following constructs: attachment, relationship quality, and relationship stability, using a longitudinal, culturally sensitive shared reality approach. We also controlled for stressful life events and explored gender differences. Moreover, we examined the stability of DoS, a hypothesis with mixed empirical longitudinal support [26]. Specifically, we sought to answer the following research questions:

Q1. Does differentiation change over time for women and men in each sample?
Q2. Do higher levels of DoS relate to better relational outcomes (lower attachment anxiety and avoidance, higher relationship quality and stability) cross-sectionally and longitudinally?
Q3. Do differences exist, regarding the two previous questions, between Spanish and U.S. couples?

## Methods

### Sample and procedure

A sample of 958 individuals (*n* = 137 couples from Spain, and *n* = 342 couples from U.S.) was used in the present study. The sample was composed of nonclinical, heterosexual couples, resulting in gender balanced numbers for both husbands and wives in both samples. All U.S. couples reported having children (because one of the selection criteria for the sample of the original research project was having a child between the ages of 11–14 in 2005), and the majority of the Spanish couples also reported having children (70.1%).

The 274 Spanish individuals in this sample had an average age of 43.19 years (*SD* = 8.73; range = 24 to 64), and their current relationship status was 85% married and 15% cohabitating (i.e., living together but not married; see S1 Table for sociodemographic data). In terms of the highest obtained level of education, 57% of participants reported graduate studies, 14% reported postgraduate education, 17% reported high school or some college, and 12% reported basic primary education. Most participants were employed (76.3%), 12% were unemployed, and the remaining reported other situations (e.g., retired, homemaker, etc.). The mean duration of couple relationships was 20.18 years (*SD* = 11.14; range = 26 months to 46 years). Race and ethnicity were not collected for the Spanish sample, a typical practice in European psychological research, and in accordance with the local IRB protocols.

The 684 U.S. participants in the sample had an average age of 43.95 years (*SD* = 5.63; range = 26 to 62), and their current relationship status was reported as 99% married, and 1% cohabitating. In terms of the highest obtained level of education, 47% of the participants reported completing graduate studies, 26% reported postgraduate education, 26% reported high school or some college, and 1% reported basic primary education. The majority of participants were employed (81%), 14% reported being a “full-time homemaker,” and the rest reported other situations (e.g., retired, unemployed, etc.). The mean duration of the relationship was 17.98 years (*SD* = 4.79; range = 2 to 35 years). The race and ethnicity of the sample was reported as follows: 82.4% Caucasian, 5.1% African American, 4.4% Hispanic, 3.5% Asian, 0.8% Native American, and 3.8% self-classified as “other”.

Participants in this study were drawn from non-clinical samples in each country, each of which received approval by the institutional review board at the institution of each principal investigator. The U.S. data came from the Flourishing Families Project (FFP), a longitudinal research project investigating family processes. The first wave of Spanish data came from a doctoral dissertation on DoS in a Spanish community sample. In the Spanish study, inclusion criteria included identifying as Spaniard and being 18 years or older, without any specific criteria about the family status or having a partner. A subsequent wave of data was collected with a specific consideration for the measures used by the FFP in order to conduct this cross-cultural study.

In both countries, some participants were recruited through convenience sampling using mailings, local phone records, and local fliers; however, the bulk of the U. S. sample was recruited using a purchased national telephone survey database (Polk Directories/InfoUSA). Families identified using the Polk Directory were randomly selected from targeted census tracts that mirrored the socio-economic and racial stratification of reports of local school districts. Spanish data included in this study was collected in 2011 (time 1) and 2018 (time 2), and U.S. data in 2007 (time 1) and 2011 (time 2). Participants were informed about the voluntary nature of the study, that the information they provided would be confidential, and that they could withdraw at any time.

### Measures

Participants completed all measures in their native language. Where noted below, a specific Spanish version of the measure was used. Relationship stability, relationship quality, and life events items were provided based on the RELATE assessment battery [35].

#### Differentiation of self

Spanish couples’ level of DoS was measured using the Spanish-Differentiation of Self Inventory (S-DSI) [36], which is composed of 26 items and two subscales: emotional cutoff (13 items) and emotional reactivity (13 items). U. S. couples reported DoS through two subscales of the Differentiation of Self Inventory (DSI) [37]: emotional reactivity (11 items) and emotional cut-off (12 items). Participants responded using a 6-point scale ranging from 1 (*Not at all true for me*) to 6 (*Very true for me*). Higher scores indicated higher levels of DoS and lower levels of emotional cutoff and emotional reactivity. In the present study, we obtained adequate to high internal consistency reliability for both samples and genders (see Table 3).

Informed by the shared reality method proposed by Busby and colleagues [13], we created a combined DoS score for the couple. First, we created an individual DoS score for everyone by summing their own emotional cutoff and emotional reactivity scores. Second, DoS scores for each partner were then summed together to form a combined DoS score to represent a shared reality of the couple’s level of differentiation.

#### Attachment

Eight items from the Revised Experiences in Close Relationships Questionnaire (ECR) [38] were used for measuring levels of anxious attachment (four items: “I am afraid that I will lose my partner’s love”; “I often worry that my partner will not want to stay with me”; “I often worry that my partner does not really love me”; “I often wish that my partner’s feelings for me were as strong as my feelings for him or her”), and levels of an avoidant attachment (four items: “I prefer not to show my partner how I feel deep down”; “I feel comfortable sharing my private thoughts and feelings with my partner”; “I find it difficult to allow myself to depend on my partner”; “I am very comfortable being close to my partner”). Spanish versions of these items were extracted from a validated version of ECR [39]. Participants were asked to score these items on a 7-point Likert response scale ranging from 1 (*strongly disagree*) to 7 (*strongly agree*), where higher scores indicated higher levels of avoidance or anxious attachment. The reliability (Cronbach’s Alpha) for this sample (see Table 3) were adequate to high.

#### Relationship stability

A three-item measure from the RELATE assessment battery was used for assessing relationship stability [40]: “How often have you thought your relationship (or marriage) might be in trouble?”; “How often have you and your partner discussed ending your relationship (or marriage)?”; and “How often have you broken up or separated and then gotten back together?” This measure used a 5-point Likert scale ranging from 1 (*never*) to 5 (*very often*). We reversed the sign of the scores so higher scores would represent more stability associated with the relationship. The Cronbach’s Alpha coefficients for both samples and for both men and women were adequate to high (see Table 3).

#### Relationship quality

Relationship quality was assessed using a modified, 4-item version of the Quality Marriage Index [25]: “We have a good relationship”; “Our relationship is strong”; “My relationship with my partner makes me happy”; and “I really feel like part of a team with my partner.” We removed the item, “My relationship with my partner is very stable,” due to similarity with the relationship stability items. A 6-point Likert scale ranging from 1 (*very strongly disagree)* to 6 *(very strongly agree)* was presented for participants to rate the items, where higher scores indicated higher perceived relationship quality. Reliability was (Cronbach’s Alpha coefficient; see Table 3) good to high for each sample and both genders.

#### Stressful life events

Ten items from Johnson were used to assess stressful life events [41]. The items were as follows: the death of a child, needing to provide care for disabled relative or friend, a residential move, a serious illness or injury in the family, the birth of a child, the death of a partner, the death of a close friend, the death of a parent, loss of a job, or a victim of crime. For this study, we produced a score summing the number of previous events each participant endorsed as having occurred during the last year. Possible scores ranged from 0 to 10, where 10 indicated the highest level of stressful life events possible. We included stressful life events at time 2 as a control variable in our models, allowing us to examine the relationship among key variables while also accounting for the sum of stressful life events up until time 2.

### Data analysis plan

We addressed missing data (Spain = 0.04%; USA = 0.50%) with multiple imputation using Bayesian analysis in Mplus 8.0. [42] Skew (Spain: -0.66 to 1.46; USA: -1.02 to 1.56) and kurtosis (Spain: -1.11 to 2.26; USA: -0.75 to 3.83) were not problematic [skewness index < |3.0|; kurtosis index < |10.0|] [43].

Conceptually, we considered our dyads distinguishable, and conducted an omnibus test of distinguishability to determine whether means, variances, and covariances differed between women and men [44, 45]. Equality constraints were placed on men’s and women’s means and variances, and we obtained a rejectable chi-square: χ^2^ (186) = 4897.52, *p* < .001. To ensure misfit of the data to the model did not occur because of the equality constraints, we systematically removed them. Without mean, variance, and covariance constraints, we obtained the following respective rejectable chi-squares: χ^2^ (220) = 9298.25, *p* < .001; χ^2^ (247) = 20844.23, *p* < .001; χ^2^ (254) = 9298.25, *p* < .001. These results support our conceptualization of distinguishability between women and men.

**Fig 1:**
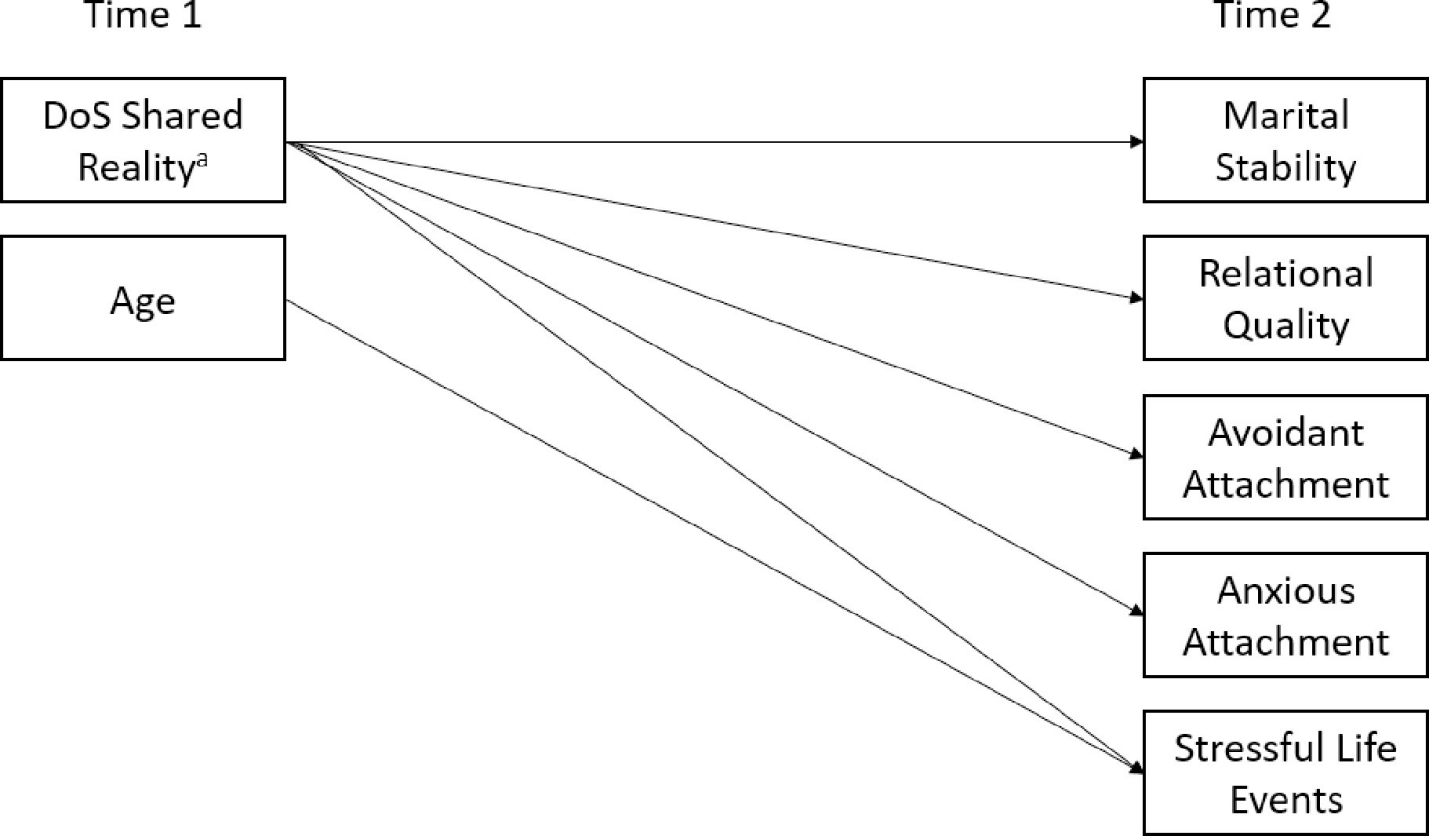
Conceptual path diagram. This figure represents the conceptual model used to analyze the association between a couple shared reality score of differentiation of self on a variety of outcomes. ^a^ DoS = differentiation of self. The shared reality score was computed by summing the scores of differentiation of self for each of the two individuals in each couple relationship. ^b^ Please note that the actual model included paths from DoS shared reality to scores on the five outcomes for men *and* women within one model. Additionally, a multigroup analysis was run therefore paths for U.S. and Spanish participants were estimated separately in two models.

The cross-sectional and longitudinal models in this study were assessed using multigroup analysis with maximum likelihood estimation in Mplus version 8.0 [42]. Fig 1 depicts the conceptual model for the longitudinal analysis. A path analytic approach allowed us to examine how couples’ combined DoS influenced key relational variables cross-sectionally and longitudinally simultaneously. The cross-sectional conceptualization was the same; however, we used the time 2 DoS shared reality and age variables. This allowed for simultaneous estimation of the analytic models for both the Spanish and U.S. samples. To determine whether significant differences existed between Spanish and U.S. couples on each of the effects, we used the “model constraint” command in Mplus to subtract Spanish couples’ estimated effects from that of U.S. couples (i.e., slope differences), and used significance testing (*p*-values) and 95% confidence intervals with 10,000 bootstrap resamples.

We created a shared reality variable for DoS to represent the couple emotional system Bowen postulated [14]. To accomplish this, we summed the men and women DoS scores at time 1 and then those at time 2, for both Spanish and U.S. couples. In a study of multiple methods of creating shared reality scores, Busby and colleagues determined that combining scores provided the most accurate representation of a shared couple reality [13]. This method informed our creation of a shared reality DoS variable for Spanish and U.S. couples.

While controlling for stressful life events and age, we regressed the variables of relationship quality, attachment, and relationship stability from time 2 on the combined DoS variable from time 2 to conduct the cross-sectional analysis. For the longitudinal analysis, we regressed relationship quality, attachment, and relationship stability from time 2 on the combined DoS variable from time 1, while controlling for stressful life events and age. In addition, to isolate the effect time 1 combined DoS had on the key relational variables, we controlled for combined DoS at time 2.

## Results

Table 1 shows bivariate correlations among DoS subscales of emotional reactivity and emotional cutoff, and Table 2 shows bivariate correlations among study variables, including the covariates of age and stress. At time 1, the DoS subscales of emotional cutoff and emotional reactivity correlated significantly for both Spanish and U.S. couples, and at time 2 for U.S. couples. At time 1, the correlations between couples in the U.S. and Spanish participant samples were statistically significant (*r*s ranging from .14 - .53). At time 2, the correlations between couples in the U.S. and Spanish participant samples, respectively, were lower, and statistically significant for the U.S. couples only.

**Table 1:** Descriptive statistics and correlations for U.S. and Spain women and men DoS subscales of emotional reactivity and emotional cutoff at Time 1 and Time 2.

										Spain		U.S.		
		1	2	3	4	5	6	7	8	*M*	SD	*M*	SD	
1	ER1: Female	-	.64**	.03	.00	.35**	.41**	-.18	-.15	3.17	0.89	3.39		1.02
2	EC1: Female	.35**	-	.08	.04	.39**	.53**	-.08	-.04	2.93	1.02	1.91		0.80
3	ER5: Female	.73**	.26**	-	.85**	.12	.12	.09	.08	2.90	1.00	2.99		1.00
4	EC5: Female	.29**	.70**	.40**	-	.15	.13	.13	.14	2.77	1.05	1.84		0.81
5	ER1: Male	.14*	.22**	.10	.18**	-	.84**	-.04	-.01	2.93	0.87	2.87		0.86
6	EC1: Male	.24**	.19**	.18**	.16**	.42**	-	-.03	.01	2.94	0.92	2.09		0.76
7	ER5: Male	.11	.18**	.12*	.15**	.69**	.33**	-	.90**	2.87	0.96	2.54		0.90
8	EC5: Male	.16**	.11*	.19**	.17**	.32**	.69**	.56**	-	2.87	1.03	1.95		0.81

Note. ER = Emotional reactivity. EC = Emotional cutoff.

*p < .05.

**p < .01

**Table 2:** Correlations among study variables for Spanish and U.S. couples.

Variables	1	2	3	4	5	6	7	8	9	10	11	12	13	14
1. Age: (W)	-	.95**	-.20*	.10	-.06	.13	.10	.21*	-.04	.01	.03	-.08	.22**	.03
2. Age: (M)	.75**	-	-.20*	.09	-.06	.15	.12	.19*	-.02	-.03	.05	-.03	.23**	.04
3. Combined Differentiation T1	-.05	.01	-	.13	.25**	-.20*	-.07	-.01	-.03	.24**	-.18*	-.10	-.05	-.13
4. Combined Differentiation T5	.03	.05	.69**	-	.40**	-.34**	-.24**	.20*	-.27**	.45**	-.30**	-.24**	.20*	-.03*
5. Relationship Quality T2: (W)	.01	-.02	.29**	.41**	-	-.55**	-.40**	.15	-.54**	.75**	-.38**	-.25**	.11	-.70*
6. Anxious Attachment T2: (W)	.03	-.02	-.29**	-.44**	-.54**	-	.43**	-.02	.32**	-.48**	.75**	.25**	-.08	.43**
7. Avoidant Attachment T2: (W)	.03	.03	-.35**	-.45**	-.68**	.58**	-	-.03	.12	-.30**	.18*	.71**	-.02	.31**
8. # Stressful events: (W)	.19**	.19**	-.01	.02	-.02	-.01	-.07	-	-.11	.19*	-.03	-.13	.13	-.08
9. Marital Stability T2: (W)	-.07	-.01	-.33**	-.41**	-.63**	.56**	.62**	.02	-	-.65**	.42**	.30**	-.03	.70**
10. Relationship Quality T2: (M)	.00	.03	.36**	.48**	.64**	-.47**	-.55**	.05	-.59**	-	-.51**	-.41**	.16	-.54**
11. Anxious Attachment T2: (M)	-.04	-.05	-.36**	-.49**	-.52**	.35**	.48**	.03	.48**	-.58**	-	.38**	-.05	.28**
12. Avoidant Attachment T2: (M)	.02	.03	-.32**	-.48**	-.53**	.39**	.45**	-.03	.45**	-.68**	.54**	-	.01	.17*
13. # of Stressful events: (M)	.07	.02	-.04	-.04	-.00	-.01	-.02	.45**	-.03	-.01	.06	.02	-	-.04
14. Marital Stability T2: (M)	-.07	-.04	-.28**	-.39**	-.54**	.42**	.48**	.02	.73**	-.70**	.60**	.55**	.04	-

*p < .05

**p < .01

***p < .001.

*Note*. Intercorrelations for Spanish participants (*n* = 137) are presented above the diagonal, and intercorrelations for U.S. participants (*n* = 342) are presented below the diagonal. (W) = Women; (M) = Men; T1 = Time 1; T2 = Time 2.

Table 3 contains descriptive statistics and mean comparisons across the samples for women and men in Spain and the U.S., with several differences emerging. Spanish women reported significantly lower relationship quality, more anxious and avoidant attachment, and more stressful life events than their U.S. counterparts. Similarly, Spanish men reported significantly greater DoS, lower relationship quality, and more avoidant attachment than U.S. men. Spanish couples had significantly lower combined differentiation scores than U.S. couples at both time points.

**Table 3:** Descriptive statistics for U.S. and Spanish couples and comparisons of means across cultures with independent samples T-tests.

		Spain (*n* = 137)			U.S. (*n* = 342)				95%*CI*	
	Variables	*M*	*SD*	*α*	*M*	*SD*	*α*	*t* (*df*)	*LL*	*UL*
*Women*										
	Differentiation T1	3.71	0.63	.76	3.50	0.56	.91	1.70(276.40)	-0.011	0.220
	Differentiation T5	3.76	0.64	.70	3.71	0.49	.92	0.95(322.74)	-0.056	0.159
	Relationship Quality T5	3.40	0.70	.88	3.87	0.87	.97	**6.14**(311.54)	0.317	0.616
	Anxious Attachment T5	2.44	1.37	.81	2.04	1.31	.92	-**2.87**(241.20)	-0.663	-0.123
	Avoidant Attachment T5	2.89	1.34	.81	2.46	1.32	.80	**-3.19**(246.86)	-0.695	-0.164
	Stressful Events T5	0.93	1.11		0.85	1.01		**-0.70**(231.71)	-0.292	0.136
	Marital Stability T5	1.65	0.62	.90	1.66	0.60	.70	0.18(242.43)	-0.112	0.134
*Men*										
	Differentiation T1	3.65	0.61	.79	3.56	0.58	.90	1.52(259.77)	-0.027	0.208
	Differentiation T5	3.85	0.66	.70	3.73	0.50	.93	**2.09**(329.66)	0.007	0.225
	Relationship Quality T5	3.39	0.65	.88	3.90	0.81	.97	**7.32**(312.51)	0.378	0.655
	Anxious Attachment T5	2.40	1.23	.73	2.25	1.33	.88	-1.21(269.08)	-0.404	0.097
	Avoidant Attachment T5	2.87	1.25	.72	2.43	1.19	.78	**-3.49**(239.84)	-0.682	-0.190
	Stressful Events T5	0.88	1.07		0.76	0.93		-1.13(221.36)	-0.325	0.087
	Marital Stability T5	1.65	0.64	.80	1.61	0.54	.68	**-**0.69(215.56)	-0.165	0.079
*Combined*										
	Differentiation T1	7.06	9.14		7.26	0.98		**2.07**(267.59)	0.010	0.381
	Differentiation T5	7.44	0.71		7.61	1.00		**2.05**(349.20)	0.010	0.328

*Note*. Two-tailed test. *CI* = Confidence interval.

Bold indicates significance at p < .05 level or greater.

We also examined whether women and men experienced changes in DoS using paired-samples *t*-tests and determined significance based on *p*-values and a 95% confidence interval range free of zero. Spanish women’s (*t*[136] = -3.20, *p* = .002, 95%*CI* = [-0.333, -0.079]) and men’s (*t*[136] = -2.74, *p* = .007, 95%*CI* = [-0.298, -0.048]) ratings of differentiation changed positively and significantly. U.S. women (*t*[341] = -5.56, *p* < .001, 95%*CI* = [-0.208, -0.099]) and men (*t*[341] = -6.69, *p* < .001, 95%*CI* = [-0.256, -0.140]) experienced significant, positive changes in differentiation also. Stated differently, on average, Spanish and U.S. women and men reported increased differentiation over time.

The *cross-sectional path analytic model* fit the data well, χ^2^ (36) = 55.755, *p* = .018, CFI = 0.99, TLI = 0.970, RMSEA = 0.048, SRMR = 0.045.034. We examined whether combined DoS at time 2 predicted outcomes at time 2 while controlling for stressful life events and age. Among Spanish and U.S. samples, we found similar concurrent (time 2) associations of combined differentiation with women’s and men’s relationship variables (see Table 4). Specifically, for both genders and countries, combined differentiation significantly and positively related to relationship quality and stability and negatively related to anxious and avoidant attachment. No significant differences emerged between Spanish and U.S. women or men when comparing these associations across cultures at time 2.

**Table 4:** Regression effects of women and men Time 2 combined differentiation of self scores on Time 2 outcome variables for couples from Spain and the U.S., and cultural differences among the effects.

	SPAIN (*n* = 137)				U.S. (*n* = 342)				Slope Differences			
			95% *CI*				95% *CI*				95% *CI*	
Outcome Variables	β	*S*.*E*.	*LL*	*UL*	β	*S*.*E*.	*LL*	*UL*	β	*S*.*E*.	*LL*	*UL*
*Women*												
Relationship Quality	0.40***	0.07	0.250	0.535	0.41***	0.05	0.306	0.498	0.04	0.09	-0.128	0.204
Relationship Stability	-0.27***	0.08	-0.418	-0.094	-0.41***	0.05	-0.505	-0.313	0.01	0.08	-0.137	0.172
Anxious Attachment	-0.34***	0.08	-0.489	-0.175	-0.44***	0.05	-0.528	-0.337	-0.08	0.18	-0.426	0.270
Avoidant Attachment	-0.24***	0.08	-0.386	-0.082	-0.45***	0.05	-0.535	-0.353	0.13	0.15	-0.159	0.452
*Men*												
Relationship Quality	0.45***	0.07	0.301	0.585	0.48***	0.05	0.381	0.563	0.03	0.08	-0.136	0.184
Relationship Stability	-0.30**	0.10	-0.479	-0.101	-0.39***	0.05	-0.483	-0.296	-0.06	0.08	-0.210	0.108
Anxious Attachment	-0.30***	0.08	-0.45	-0.122	-0.49***	0.04	-0.562	-0.415	0.14	0.16	-0.165	0.453
Avoidant Attachment	-0.24**	0.08	-0.394	-0.069	-0.48***	0.05	-0.564	-0.383	0.16	0.15	-0.141	0.469

*Note*. Slope differences tested whether country functioned as a mediator. Β = Unstandardized estimates; S.E. = Standard error; CI = Confidence interval.

*p < .05

**p < .01

***p < .001.

The *longitudinal path analytic model* also fit the data well, χ^2^ (40) = 66.928, *p* = .044, CFI = 0.994, TLI = 0.976, RMSEA = 0.040, SRMR = 0.028. We tested whether shared DoS at time 1 predicted outcomes at time 2, while controlling for stressful life events, age, and combined DoS at time 2 (see Table 5). Among Spanish couples, combined differentiation positively predicted women’s and men’s relationship quality, and negatively predicted their anxious attachment. Among U.S. couples, combined differentiation positively predicted women’s and men’s relationship quality and stability, and negatively predicted anxious and avoidant attachment. Differences between Spanish and U.S. women emerged but not men. Women in Spain and the U.S. significantly differed on the effects that combined differentiation at time 1 had on relationship stability and avoidant attachment at time 2.

**Table 5:** Regression effects of women and men Time 1 combined differentiation of self scores on Time 2 outcome variables for couples from Spain and the U.S., and cultural differences among the effects.

	SPAIN (*n* = 137)				U.S. (*n* = 342)				Slope Differences			
			95% *CI*				95% *CI*				95% *CI*	
Outcome Variables	β	*S*.*E*.	*LL*	*UL*	β	*S*.*E*.	*LL*	*UL*	β	*S*.*E*.	*LL*	*UL*
*Women*												
Relationship Quality	0.25**	0.08	0.088	0.406	0.29***	0.06	0.175	0.394	-0.06	0.08	-0.221	0.100
Relationship Stability	-0.03	0.08	-0.172	0.124	-0.33***	0.05	-0.421	-0.219	0.18**	0.06	0.061	0.306
Anxious Attachment	-0.20*	0.08	-0.351	-0.033	-0.29***	0.05	-0.393	-0.184	0.10	0.15	-0.200	-0.382
Avoidant Attachment	-0.07	0.09	-0.235	0.097	-0.35***	0.05	-0.450	-0.242	0.37**	0.15	0.081	0.647
*Men*												
Relationship Quality	**0.24** **	0.08	0.081	0.386	0.36***	0.05	0.253	0.452	-0.13	0.08	-0.271	0.028
Relationship Stability	-0.13	0.07	-0.266	0.009	-0.28***	0.05	-0.380	-0.176	0.06	0.06	-0.052	0.170
Anxious Attachment	**-0.18** *	0.08	-0.330	-0.026	-0.36***	0.05	-0.441	-0.262	0.24	0.13	-0.021	0.484
Avoidant Attachment	-0.10	0.08	-0.258	0.064	-0.32***	0.05	-0.422	-0.213	0.26*	0.13	-0.003	0.507

*Note*. Slope differences tested whether country functioned as a mediator. Β = Unstandardized estimates; S.E. = Standard error; CI = Confidence interval.

*p < .05

**p < .01

***p < .001.

## Discussion

In this study, we sought to answer important questions about the impact of differentiation on couple relationship variables, accounting for the possibility of gender and cultural differences and controlling for age and stressful life events. Perhaps the most valuable contribution of this project is the confirmation of the relevance of DoS in relationship functioning through two different cultures and with longitudinal data, demonstrating that DoS may have long-term benefit for couple relationships. Specifically, findings from our study suggest that a higher level of DoS in the couple predicts a perception of increased relationship quality in women and men (both in the cross-sectional and longitudinal models) and a reduction of anxious attachment levels, as well as a positive effect on the perception of relationship stability (both cross-sectionally and longitudinally for U.S. women and men, and only cross-sectionally for Spanish women and men). Some significant differences by gender arise in avoidant attachment and stability in the longitudinal model.

### Changes in DoS over time

Regarding the first research question, this study shows evidence that women and men from both cultural groups displayed increases in DoS over time. The variability of DoS within a person might best be understood under Bowen’s conceptualization of the pseudo self and solid self [14]. The pseudo self is characterized by more emotional reactivity and instability, and is more prone to influence from stressors and to change based on perceived context. Individuals with lower levels of differentiation are conceptualized to more commonly function based on the pseudo self. Conversely, the solid self is characterized by more consistent conviction and more stability over time, and individuals with higher levels of DoS are postulated to operate from a place of the solid self [14, 29]. Handley and colleagues found more stability (less change) in DoS over time with their sample [26], while the current study found a significant increase in DoS over time in both men and women and in both cultures. Notably, Handley and colleagues used yearly intervals in their assessment of changes in DoS [26], while the current study had a much longer interval between the two time points assessed (four years in the U.S. sample, seven years in the Spanish sample). It is possible that in the current sample, the greater amount of time between assessments of DoS was an important factor in forming a more solid self, particularly during a time of stressful events. Moreover, as Handley and colleagues [26] mentioned, it is uncertain whether this would reflect a change in the pseudo self, or a change in the solid self. In other words, because our samples increased significantly in DoS over such long periods, we postulate that the solid self could change over time.

The results of this study also show that the correlation between DoS scores at time 1 and time 2 is much stronger in the U.S. sample than in the Spanish sample (see Tables 1 and 2). This might be due to a larger U.S. sample (342 couples) than Spanish sample (137 couples), enabling a more sensitive analytical approach, but we could not identify a theoretical explanation as to why one culture might show more or less stability in DoS than another. Both samples were similar in age (Spain *M*_*age*_ = 43.19; U.S. *M*_*age*_ = 43.95), ruling out the possibility of greater susceptibility of a younger sample to change because their DoS levels have not solidified yet.

### Cross-sectional and longitudinal associations between DoS and outcome variables

Our second research question examined whether cross-sectional and longitudinal associations between DoS and the various outcome variables existed.

#### Cross-sectional model

DoS was significantly associated with outcome variables within each wave. DoS was found to be negatively associated with anxious and avoidant attachment among women and men in both cultural groups. These findings coincide with other studies showing that increased DoS is inversely related to anxious and avoidant attachment [21, 22], and are coherent with our study hypothesis, BFST theory, and prior empirical findings. From an attachment perspective [19], individuals who know how to more effectively regulate their own emotions and avoid emotional flooding are less likely to perceive threats to their significant attachments, thereby displaying more secure attachment in their relationships.

Consistent with previous literature [12, 24, 34, 36, 46], DoS was positively associated with relationship quality for both Spanish and U.S. women and men. Moreover, DoS was significantly associated with a better perception of relationship stability in all groups (genders and cultures).

According with family systems theory, DoS could be central to long-term mutuality and intimacy in romantic couple relationships [14]. DoS is related to the capacity to maintain flexible interpersonal and intrapersonal self-regulation strategies, even under stress, keeping a connection with significant others (in particular with a partner and other close family relatives), and this capacity is linked with the family of origin patterns and experiences [9, 14, 37]. These results lend support to the Bowen’s notion that levels of DoS in the couple system have a significant role on the couple relationship functioning.

#### Longitudinal model

The central research question and most valuable contribution to the literature lies within the longitudinal impact of combined couple differentiation on the outcome variables. A recent scoping review, conducted by Calatrava and colleagues [46], pointed out more longitudinal studies in the field of DoS are needed. Specifically, among the 56 empirical studies reviewed that tested the potential positive effect of higher levels of DoS on the couple relationship, only two of them presented longitudinal data with a sample of more than 300 participants [10, 31, 47]. This is the first study where a comparison between two different cultures has been conducted, and with a sample size near 1,000 participants (N = 958). Additionally, this is the first study, to our knowledge, to analyze the longitudinal effects of DoS on anxious and avoidant attachment.

Our results indicate that higher levels of differentiation in a couple imply a greater perception of relationship quality and lower anxious attachment in both genders and countries. In other words, DoS has some long-term impact on different aspects of the couple relationship and attachment. While a large body of literature exists focusing on the cross-cultural effects of DoS on relationship quality, longitudinal studies remain scarce [46]. Our longitudinal results are consistent with the majority of the previous studies where this positive relationship was found.

In addition, longitudinal analyses offer us other results that vary according to culture. Specifically, in the U.S. participants but not the Spanish ones, higher levels of differentiation were related to lower avoidant attachment levels and higher perception of relationship stability.

Beginning with the attachment outcomes, higher levels of differentiation predicted lower avoidant attachment among U.S. women and men, but not among Spanish women and men.

This difference in the results between the samples from Spain and the U.S. could be due, perhaps, to the smaller sample size of the Spanish group. Future studies with larger sample sizes may seek to replicate this result. However, despite the smaller size of the Spanish sample, the relationship between DoS and anxious attachment is robust. That is, there could be some reason why the impact of the levels of differentiation on attachment was greater in the anxious dimension than in the avoidant one. This finding is consistent with some previous studies conducted with Italian and U.S. participants [17, 18]. Researchers in those studies used the same attachment measurement instrument as in the present study. In our results, we observed one of two things: either (a) the association between anxious attachment and differentiation remains significant when the avoidant attachment ceases to be so, or (b) the statistical indices of this association are higher when we link differentiation and anxious attachment than when we link avoidant attachment. This tendency is present also in general in the current study, both in the cross-sectional and longitudinal analyses. With the exception of U.S. women, the relationship between differentiation and anxious attachment presents more robust links than that with avoidant attachment.

The data offered by previous studies led us to consider that the difference found may not actually be a manifestation of a cultural difference between U.S. and Spain in the way in which the level of differentiation effects avoidant attachment, but rather the relationship between avoidant attachment and differentiation level more universally. As suggested by Lampis and Cataudella [17], it could be that components of differentiation, such as a lower ability to take an I-position in relationships and more challenges in maintaining emotional closeness in disagreement or under stress, may be more closely linked with attachment avoidance. Perhaps this relationship between differentiation and attachment avoidance is further minimized in collectivistic cultures where closeness is of even greater cultural importance, such as Spain and Italy. Future studies should continue to explore the subscale of differentiation in relation to different attachment styles to further understand how differentiation is useful toward healthy and secure adult attachment.

Finally, in terms of the longitudinal effects of DoS on relationship stability, higher levels of combined couple differentiation were linked with more stability in relationships for U.S. men and women with a lower possibility of ruptures over time. These results were not found among Spanish men and women. There are several possible explanations for these findings. First, it is notable that the Spanish sample size is smaller than the U.S. sample. This is notable because rates of divorce are also higher in Spain relative to the U.S. (61% versus 53% respectively) [48]. Further, rates of divorce among Spanish couples are the highest when members of the couple relationship are in their 40’s and the mean age of the Spanish sample in this study is 43 years [48]. Together, these statistics point to the potential of relationship stability being overall lower for the Spanish sample versus the U.S. sample. Second, the time lag between T1 and T2 is also different across the two samples, with the Spanish time to follow-up being, on average, three years longer (almost double the follow up time for the U.S. sample). It could be that following couples for a longer period of time also gives more room for couple relationship instability to occur; we note this as a potential confound and limitation of the current study. A second potential reason may relate to the differences among the samples in the percent of married versus unmarried (although involved in a couple relationship) participants (see S1 Table). The U.S. sample has a lower percentage (around 1%) of unmarried couples than the Spanish sample (approximately 14%). This could cause differences in the perception of relationship stability across samples from the two countries.

The findings that combined couple differentiation is not linked with avoidant attachment and relationship stability for Spanish couples could be further explained by cultural differences. Spain is a country with stronger collectivistic and familistic culture than U.S. [49]. While the underlying reasons for these differences are unclear, perhaps the unique influence of DoS is less distinct among members of a more collectivist culture characterized by more connection and interdependence [50, 51].

### Differences in the effects of DoS on outcome variables between groups

In response to the third and final research question, it should be noted that most of the results were similar between the Spanish and U.S. men and women, however, there were some noticeable differences. We found similar patterns between Spanish and U.S. men in the current study; however, we found significant difference between Spanish and U.S. women regarding the effects of combined differentiation at time 1 on avoidant attachment at time 2 (see Table 5). The other significant difference between these two groups of women emerged in the longitudinal effect of differentiation on relationship stability. In both of these cases, U.S. women were more prone to positive effects of differentiation on attachment and relationship outcomes than the Spanish women.

The fact that higher DoS at time 1 was not related to lower avoidant attachment at time 2 in women could be explained by a cross-interaction between sex/gender and culture. In Spanish culture, women have gained a power space and autonomy more recently than have U.S. women, and perhaps this increases a tendency toward independence from the partner. The samples in the current study are comprised of middle-aged women (our samples from both countries have a mean age of 43 years), with couple relationship satisfaction levels lower among Spanish women. This may occur due to evolving Spanish culture and gender roles wherein Spanish women seek to preserve an autonomous position from their partners, whereas, over the last decade, Spanish men have sought enhanced connection with their partners.

Regarding stability, as mentioned prior, Spain may present a cultural context where couples have a heightened awareness of divorce. Research has shown two important aspects that influence divorce. First, women are more likely to initiate divorce [52]. Second, the financial contribution of men and women in marriage has a relevant impact on satisfaction and divorce, where financial dependency of either partner could decrease divorce ideation [53, 54]. Additionally, in the current study, 14% (approximately) of the Spanish couples have no legal recognition of their relationship, and 16.8% of Spanish women reported themselves as homemakers compared to 26% of U.S. women (see S1 Table). Perhaps these two variables create a difference of the perception of the couple relationship, to which women may be particularly responsive.

### Limitations

This study it is not without limitations, despite offering a significant contribution to the literature by disentangling the effects of DoS on couple relationship outcomes and exploring differences between Spanish and U.S. couples. First, there is a difference in the time (years) in which the data was collected between the two samples along with differences in the time between each wave (Spanish sample = 7 years, U.S. sample = 4 years). In terms of psychometrics, there were slight differences between the S-DSI (26 items) and DSI (23 items), which were used to measure DoS for each group. This eliminated the option to perform a group comparison analysis testing constrained and unconstrained models to find differences at the measurement level [54]. Although this study included a longitudinal component, there were only two time points available in both samples, making it difficult to predict outcomes using a growth curve analysis (requiring three or more time points). Studies that involve more time points would be valuable, as they would permit researchers to better understand more complex changes in DoS and relational outcomes over time (e.g., inclines and declines or curvilinear changes in relationship quality). It is also important to note that all participants were collected as a study of non-clinical and (presumably) healthier relationships, a probable bias in regard to some findings (e.g., greater variabilities in DoS and outcome scores would be likely across a sample of clinical couples).

Finally, all variables in our study were self-reported, leading to the potential for bias introduced by mono-method limitations. In particular, we used a shared reality method informed by that of Busby et al., but lacked female reports of her partner’s DoS, and vice versa [13]. Self- and other-reports of DoS may help further elucidate a shared reality of the couple’s emotional system as Bowen postulated. Our analysis is a step in that direction. Researchers may utilize other methods to analyze dyadic data including the APIM approach, which provides researchers a method to account for the interdependence of dyadic data. Additionally, although at time 2 correlations for Spanish couples were low, we posited that differing views of their own DoS can still combine to create a shared reality within the relationship.

### Future research and therapeutic implications

#### Research directions

This study aligns with previous findings suggesting that DoS is relevant for relationship functioning [12, 24, 34, 46] and adds the most consistent contribution to date confirming this positive relationship between differentiation and higher relationship quality. Although previous studies, which almost exclusively use cross-sectional data and omit dyadic data, generally indicate that DoS has an influence on relationship functioning regardless of culture, our study contributes with a larger sample and consistent results across cross-sectional and longitudinal data, Spanish and U.S. couples, and men and women.

Future studies should continue this direction and pay particular attention to the mechanism behind the confirmed relationship between DoS and relationship functioning and consider two key points: the need for longitudinal designs and the measurement of DoS. In regards to the first point, while there are dozens of cross-sectional studies on DoS and relationship functioning, longitudinal studies remain scarce. To our knowledge, only two samples contain longitudinal data, one from the U.S. and the other Spain. Both of these have been used in the current study. This limits any potential interpretations, as no other longitudinal data exists for comparison. The longitudinal data about DoS from the U.S. comes from the Flourishing Families Project (FFP), which has been used in three prior and relevant published studies [10, 26, 31]. This clearly underscores the need to develop new projects regarding DoS and relationship functioning using longitudinal designs.

Regarding the second point, the results obtained in this study are conditioned by a self-reported measure of DoS. Some researchers, both experts on DoS and on relationship functioning [55, 56], have raised the need to advance the study of relational processes, not only towards longitudinal designs, but also towards the use of more complex and complete measures, such as the combination of psychophysiological measures of differentiation, semi-structured interviews, or standardized observational elements to assess relationship adjustment. Although Bowen posited couples have similar DoS levels, this remains an empirical question needing continued investigation. Future studies might directly test the shared reality hypothesis of couple differentiation and the best way to obtain such a score. This study provides a step forward in understanding and hypothesizing about a shared reality of differentiation between a couple and its predictive power in relationship functioning. Future studies should also focus on the development of new, non-self-reported measures of DoS that complement the self-reported questionnaire method.

#### Clinical implications

Considering the empirical results from this study, we identified important therapeutic implications for professionals who conduct couples therapy. We found numerous significant associations between DoS and relevant variables for relationship functioning in the two cultures and for men and women. Considering that Bowen already stated that the levels of differentiation can be modified through psychotherapy [14], we propose that clinical intervention can improve the levels of differentiation and, with it, the relationship adjustment. Therapeutic interventions aimed at promoting DoS, that is, enhancing the couples´s capacity to self-regulate and balance connectedness and separateness, will enhance their relationship satisfaction. Our results also indicate that psychotherapeutic techniques that promote a higher level of DoS can reduce anxious attachment patterns and improve the couple’s capacity to diminish the use of hyperactivating behaviors aimed to force partners to give more support and attention when facing relational conflicts.

Therefore, we urge clinicians and psychotherapists who work with couples to consider the relevance of DoS and the Bowen Family Systems Theory to their theoretical frameworks, and to enhance their clinical evaluations and interventions.

In conclusion, our work is the first longitudinal study, to our knowledge, that includes dyadic data from two countries. The results provide greater clarity on how differentiation of self relates to important relational variables in two cultures and over time. We anticipate relationship and intervention researchers will benefit by integrating these results into their study and practice.

## Supporting information

S1 Table Sociodemographic information for women and men from Spain and U.S. (DOCX)

S1 File Codebook Spain USA. (DOCX)

S2 File Mplus input Syntax_Cross sectional. (DOCX)

S3 File Mplus input Syntax_Longitudinal. (DOCX)
