# Supplementary material for: Differentiation of self and relationship attachment, quality, and stability: A path analysis of dyadic and longitudinal data from Spanish and U.S. couples
Source: PLoS One. 2023 Mar 2;18(3):e0282482. doi: 10.1371/journal.pone.0282482 (PMC9980780; doi:10.1371/journal.pone.0282482)
Supplement: S1 Table — (DOCX) [file pone.0282482.s001.docx]

| Table S.1. *Sociodemographic Information for Women and Men from Spain and U.S.* | | | | | | |
| --- | --- | --- | --- | --- | --- | --- |
|  |  | Spain (n = 137) | |  | U.S. (n = 342) | |
|  | | Women | Men |  | Women | Men |
| Variables | | % | % |  | % | % |
| *Education* | |  |  |  |  |  |
|  | Less than High School | 8.8 | 12.4 |  | 1.5 | 0 |
|  | High School | 4.4 | 8.8 |  | 5.3 | 5.3 |
|  | Some college | 12.4 | 9.5 |  | 21.3 | 20.5 |
|  | Associate's | 9.5 | 7.3 |  | 5.6 | 4.1 |
|  | Bachelor's | 50.4 | 46.7 |  | 42.4 | 40.9 |
|  | Master's Degree | 12.4 | 15.3 |  | 18.1 | 17 |
|  | Advanced Degree (JD, Ph.D, PsyD, etc) | 0.0 | 0.0 |  | 5.8 | 12.3 |
|  |  |  |  |  |  |  |
| *Work Status* | |  |  |  |  |  |
|  | Working now, employed by someone else | 67.2 | 85.4 |  | 49.7 | 73.7 |
|  | Self-employed | 0.0 | 0.0 |  | 18.1 | 20.5 |
|  | Temporarily laid off | 0.0 | 0.0 |  | 0.3 | 0 |
|  | Unemployed, looking for work | 13.1 | 10.9 |  | 1.5 | 0.3 |
|  | Full-time homemaker | 16.8 | 0.0 |  | 26 | 1.2 |
|  | Permanently disabled, unable to work | 1.5 | 0.7 |  | 0.3 | 0.3 |
|  | Retired | 1.5 | 2.9 |  | 0 | 0.6 |
|  | Studying, not working | 0.0 | 0.0 |  | 0.3 | 1.2 |
|  | Other | 0.0 | 0.0 |  | 3.8 | 2.3 |
|  | |  |  |  |  |  |
| *Marital Status (Time 1)* | |  |  |  |  |  |
|  | Cohabiting | 0.0 | 0.0 |  | 0.9 | 0.9 |
|  | Married | 85.4 | 84.7 |  | 99.1 | 98.8 |
|  | Single, Never Married | 13.9 | 14.6 |  | 0.0 | 0.3 |
|  | Married/Separated | 0.7 | 0.0 |  | 0.0 | 0.0 |
|  | Divorced (not remarried) | 0.0 | 0.7 |  | 0.0 | 0.0 |
|  | |  |  |  |  |  |
| *Marital Status (Time 2)* | |  |  |  |  |  |
|  | Cohabiting | 0.0 | 0.0 |  | 0.9 | 0.6 |
|  | Married | 86.9 | 86.1 |  | 98.8 | 99.1 |
|  | Single, Never Married | 13.1 | 13.1 |  | 0.0 | 0.3 |
|  | Divorced (not remarried) | 0.0 | 0.7 |  | 0.0 | 0.0 |
