# Supplementary material for: Differentiation of self and relationship attachment, quality, and stability: A path analysis of dyadic and longitudinal data from Spanish and U.S. couples
Source: PLoS One. 2023 Mar 2;18(3):e0282482. doi: 10.1371/journal.pone.0282482 (PMC9980780; doi:10.1371/journal.pone.0282482)
Supplement: S1 File — (DOCX) [file pone.0282482.s002.docx]

Participants in this study were drawn from prior studies conducted in each country with approval from the institutional review board of each primary investigator. The Flourishing Families Project (FFP), a longitudinal research project examining family processes, provided data on U.S. couples. Spanish data (First Wave) came from a doctoral dissertation on differentiation of self. A follow-up wave of data was collected, with a focus on variables collected in the FFP project to examine couples cross-culturally.

**DATA DOCUMENTATION FOR OPENICPSR #** **181141**

*Differentiation of Self and Relationship Attachment, Quality and Stability:*

*A Path Analysis of Dyadic and Longitudinal Data from Spanish and U.S. Couples*

**Variable Names:** For ease of identification in the codebook variables were named. As a reference, all changes are noted below.

| **SPSS Variable Name** | **Codebook Variable Name** | **Mplus Variable Name** |
| --- | --- | --- |
| ID |  | id |
| Country | A1 | Ctry |
| Age_Female | A2 | AGEf1 |
| F_sch_1 | A3 | - |
| F_wrk1_1 | A4 | - |
| F_mar_1 | A5 | - |
| F_mar_2 | A6 | - |
| M_age_1 | A7 | AGEm1 |
| M_sch_1 | A8 | - |
| M_wrk1_1 | A9 | - |
| M_mar_1 | A10 | - |
| M_mar_2 | A11 | - |
| F_att1_2 | B1a | - |
| F_att2_2 | B1b | - |
| F_att3_2 | B1c | - |
| F_att4_2 | B1d | - |
| F_att5_2 | B1e | - |
| F_att6_2 | B1e | - |
| F_att7_2 | B1f | - |
| F_att8_2 | B1g | - |
| F_att9_2 | B1h | - |
| F_ins1_2 | B2a | - |
| F_ins2_2 | B2b | - |
| F_ins3_2 | B2c | - |
| F_rel1_2 | B3a | - |
| F_rel3_2 | B3c | - |
| F_rel4_2 | B3d | - |
| F_rel5_2 | B3e | - |
| F_df1_1 | B4a | - |
| F_df2_1 | B4b | - |
| F_df3_1 | B4c | - |
| F_df4_1 | B4d | - |
| F_df5_1 | B4e | - |
| F_df6_1 | B4f | - |
| F_df7_1 | B4g | - |
| F_df8_1 | B4h | - |
| F_df10_1 | B4j | - |
| F_df11_1 | B4k | - |
| F_df12_1 | B4l | - |
| F_df13_1 | B4m | - |
| F_df14_1 | B4n | - |
| F_df15_1 | B4o | - |
| F_df16_1 | B4p | - |
| F_df17_1 | B4q | - |
| F_df18_1 | B4r | - |
| F_df19_1 | B4s | - |
| F_df20_1 | B4t | - |
| F_df21_1 | B4u | - |
| F_df22_1 | B4v | - |
| F_df23_1 | B4w | - |
| F_df1_2 | B5a | - |
| F_df2_2 | B5b | - |
| F_df3_2 | B5c | - |
| F_df4_2 | B5d | - |
| F_df5_2 | B5e | - |
| F_df6_2 | B5f | - |
| F_df7_2 | B5g | - |
| F_df8_2 | B5h | - |
| F_df9_2 | B5i | - |
| F_df10_2 | B5j | - |
| F_df11_2 | B5k | - |
| F_df12_2 | B5l | - |
| F_df13_2 | B5m | - |
| F_df14_2 | B5n | - |
| F_df15_2 | B5o | - |
| F_df16_2 | B5p | - |
| F_df17_2 | B5q | - |
| F_df18_2 | B5r | - |
| F_df19_2 | B5s | - |
| F_df20_2 | B5t | - |
| F_df21_2 | B5u | - |
| F_df22_2 | B5v | - |
| F_df23_2 | B5w | - |
| M_att1_2 | C1a | - |
| M_att2_2 | C1b | - |
| M_att3_2 | C1c | - |
| M_att4_2 | C1d | - |
| M_att5_2 | C1e | - |
| M_att6_2 | C1f | - |
| M_att7_2 | C1g | - |
| M_att8_2 | C1h | - |
| M_ins1_2 | C2a | - |
| M_ins2_2 | C2b | - |
| M_ins3_2 | C2c | - |
| M_rel1_2 | C3a | - |
| M_rel3_2 | C3c | - |
| M_rel4_2 | C3d | - |
| M_rel5_2 | C3e | - |
| M_df1_1 | C4a | - |
| M_df2_1 | C4b | - |
| M_df3_1 | C4c | - |
| M_df4_1 | C4d | - |
| M_df5_1 | C4e | - |
| M_df6_1 | C4f | - |
| M_df7_1 | C4g | - |
| M_df8_1 | C4h | - |
| M_df9_1 | C4i | - |
| M_df10_1 | C4j | - |
| M_df11_1 | C4k | - |
| M_df12_1 | C4l | - |
| M_df13_1 | C4m | - |
| M_df14_1 | C4n | - |
| M_df15_1 | C4o | - |
| M_df16_1 | C4p | - |
| M_df17_1 | C4q | - |
| M_df18_1 | C4r | - |
| M_df19_1 | C4s | - |
| M_df20_1 | C4t | - |
| M_df21_1 | C4u | - |
| M_df22_1 | C4v | - |
| M_df23_1 | C4w | - |
| M_df1_2 | C5a | - |
| M_df2_2 | C5b | - |
| M_df3_2 | C5c | - |
| M_df4_2 | C5d | - |
| M_df5_2 | C5e | - |
| M_df6_2 | C5f | - |
| M_df7_2 | C5g | - |
| M_df8_2 | C5h | - |
| M_df9_2 | C5i | - |
| M_df10_2 | C5j | - |
| M_df11_2 | C5k | - |
| M_df12_2 | C5l | - |
| M_df13_2 | C5m | - |
| M_df14_2 | C5n | - |
| M_df15_2 | C5o | - |
| M_df16_2 | C5p | - |
| M_df17_2 | C5q | - |
| M_df18_2 | C5r | - |
| M_df19_2 | C5s | - |
| M_df20_2 | C5t | - |
| M_df21_2 | C5u | - |
| M_df22_2 | C5v | - |
| M_df23_2 | C5w | - |
| F_ANX_2 | D1 | ANXf2 |
| F_AVO_2 | D2 | AVOf2 |
| F_INS_2 | D3 | INSf2 |
| F_RQ_2 | D4 | RQF2 |
| F_DSI_1 | D5 | - |
| F_DSI_2 | D6 | - |
| F_strs_2 | D7 | STRf2 |
| M_ANX_2 | D8 | ANXm2 |
| M_AVO_2 | D9 | AVOm2 |
| M_INS_2 | D10 | INSm2 |
| M_RQ_2 | D11 | RQm2 |
| M_DSI_1 | D12 | - |
| M_DSI_2 | D13 | - |
| M_strs_2 | D14 | STRm2 |
| DSIFM_1 | D15 | DSIFM1 |
| DSIFM_2 | D16 | DSIFM2 |

A. Demographic Variables

A1. Country of Origin

Data collection occurred separately in each country (i.e., Spain and the United States [USA]). Value (0 = USA; 1 = Spain assignment occurred automatically based on where participants completed the data.

**Female Demographic Data**

A2. Age of participant (FEMALE)

A3. Highest education level (FEMALE)

1 Less than high School

2 High School

3 Some College

4 Associate’s Degree

5 Bachelor’s Degree

6 Master’s Degree

7 Advanced Degree (JD, Ph.D, PsyD, etc)

A4. What is your current work situation? (FEMALE)

1. Working now, Employed by someone else
2. Self-employed
3. Temporarily laid off
4. Unemployed, looking for work
5. Full-time homemaker
6. Retired
7. Permanently disabled, unable to work
8. Studying, not working
9. Other (Specify)

A5. What is your current marital status [TIME 1]? (FEMALE)

1. Single, never married
2. Married
3. Married/Separated
4. Divorced
5. Cohabiting
6. Widowed
7. Not cohabiting but in a committed relationship

A6. What is your current marital status [TIME 2]? (FEMALE)

1. Single, never married
2. Married
3. Married, but currently separated
4. Divorced (and not remarried)
5. Cohabiting (living together)
6. Widowed (and not remarried)
7. Not cohabiting, but in a committed relationship

**Male Demographic Data**

A7. Age of participant (MALE)

A8. Highest education level (MALE)

1 Less than high School

2 High School

3 Some College

4 Associate’s Degree

5 Bachelor’s Degree

6 Master’s Degree

7 Advanced Degree (JD, Ph.D, PsyD, etc)

A9. What is your current work situation? (MALE)

1. Working now, Employed by someone else
2. Self-employed
3. Temporarily laid off
4. Unemployed, looking for work
5. Full-time homemaker
6. Retired
7. Permanently disabled, unable to work
8. Studying, not working
9. Other (Specify)

A10. What is your current marital status [TIME 1]? (MALE)

1. Single, never married
2. Married
3. Married/Separated
4. Divorced
5. Cohabiting
6. Widowed
7. Not cohabiting but in a committed relationship

A11. What is your current marital status [TIME 2]? (MALE)

1. Single, never married
2. Married
3. Married, but currently separated
4. Divorced (and not remarried)
5. Cohabiting (living together)
6. Widowed (and not remarried)
7. Not cohabiting, but in a committed relationship

SECTION B PERTAINS TO **FEMALE** VARIABLES

B. Attachment, Relationship Stability,

Relationship Quality, and Differentiation of Self [TIME 2]

B1. Read each statement and decide how much you agree or disagree with it.

B2. How often is this true of your relationship?

B3. How much do you agree with these statements?

B4. In this section, we would like to know more about your emotions. Please read each statement carefully and decide how true it is for you. [TIME 1]

B5. In this section, we would like to know more about your emotions. Please read each statement carefully and decide how true it is for you. [TIME 2]

SECTION C PERTAINS TO **MALE** VARIABLES

C. Attachment, Relationship Stability,

Relationship Quality, and Differentiation of Self [TIME 2]

C1. Read each statement and decide how much you agree or disagree with it.

C2. How often is this true of your relationship?

C3. How much do you agree with these statements?

C4. In this section, we would like to know more about your emotions. Please read each statement carefully and decide how true it is for you. [TIME 1]


C5. In this section, we would like to know more about your emotions. Please read each statement carefully and decide how true it is for you. [TIME 2]

D. Average, Sum, and Combination of Created Variables

(SPSS syntax for variable creation)

**FEMALE VARIABLES**

D1. Averaged Anxious Attachment Subscale: Time 2 [FEMALE]

COMPUTE F_ANX_2 = SUM((F_att1_2 + F_att2_2 + F_att3_2 + F_att4_2)/4).

D2. Averaged Avoidant Attachment Subscale: Time 2 [FEMALE]

COMPUTE F_AVO_2 = SUM((F_att5_2 + F_att6_2 + F_att7_2 + F_att8_2)/4).

D3. Averaged Marital Instability Scale: Time 2 [FEMALE]

COMPUTE F_INS_2 = SUM((F_ins1_2 + F_ins2_2 + F_ins3_2)/3).

D4. Averaged Relationship Quality Index: Time 2 [FEMALE]

COMPUTE F_RQ_2 = SUM((F_rel1_2 + F_rel3_2 + F_rel4_2 + F_rel5_2)/5).

*Note.* Item F_rel2_2 removed due to similarity to Marital Instability items.

D5. Averaged Differentiation of Self Inventory: Time 1 [FEMALE]

COMPUTE F_DSI_1 = SUM((F_df1_1 + F_df2_1 + F_df3_1 + F_df4_1 + F_df5_1 + F_df6_1 + F_df7_1 + F_df8_1 + F_df9_1 + F_df10_1 + F_df11_1 + F_df12_1 + F_df13_1 + F_df14_1 + F_df15_1 + F_df16_1 + F_df17_1 + F_df18_1 + F_df19_1 + F_df20_1 + F_df21_1 + F_df22_1 + F_df23_1)/23).

D6. Averaged Differentiation of Self Inventory: Time 2 [FEMALE]

COMPUTE F_DSI_2 = SUM((F_df1_2 + F_df2_2 + F_df3_2 + F_df4_2 + F_df5_2 + F_df6_2 + F_df7_2 + F_df8_2 + F_df9_2 + F_df10_2 + F_df11_2 + F_df12_2 + F_df13_2 + F_df14_2 + F_df15_2 + F_df16_2 + F_df17_2 + F_df18_2 + F_df19_2 + F_df20_2 + F_df21_2 + F_df22_2 + F_df23_2)/23).

D7. Summed Number of Stressful Life Events in the Last Year [FEMALE]

Participants chose among the list of 10 stressful events and indicated whether it happened in the last year.

All selected stressful events were summed to create a total score.

*Note*. Due to differences in the two datasets (i.e., Spanish, and Flourishing Families Project ) only stressful life events in the last year were used, as opposed to including those that happened more than a year ago also.

**MALE VARIABLES**

D8. Averaged Anxious Attachment Subscale: Time 2 [MALE]

COMPUTE M_ANX_2 = SUM((M_att1_2 + M_att2_2 + M_att3_2 + M_att4_2)/4).

D9. Averaged Avoidant Attachment Subscale: Time 2 [MALE]

COMPUTE M_AVO_2 = SUM((M_att5_2 + M_att6_2 + M_att7_2 + M_att8_2)/4).

D10. Averaged Marital Instability Scale: Time 2 [MALE]

COMPUTE M_INS_2 = SUM((M_ins1_2 + M_ins2_2 + M_ins3_2)/3).

D11. Averaged Relationship Quality Index: Time 2 [MALE]

COMPUTE M_RQ_2 = SUM((M_rel1_2 + M_rel3_2 + M_rel4_2 + M_rel5_2)/5).

*Note.* Item F_rel2_2 removed due to similarity to Marital Instability items.

D12. Averaged Differentiation of Self Inventory: Time 1 [MALE]

COMPUTE M_DSI_1 = SUM((M_df1_1 + M_df2_1 + M_df3_1 + M_df4_1 + M_df5_1 + M_df6_1 + M_df7_1 + M_df8_1 + M_df9_1 + M_df10_1 + M_df11_1 + M_df12_1 + M_df13_1 + M_df14_1 + M_df15_1 + M_df16_1 + M_df17_1 + M_df18_1 + M_df19_1 + M_df20_1 + M_df21_1 + M_df22_1 + M_df23_1)/23).

D13. Averaged Differentiation of Self Inventory: Time 2 [MALE]

COMPUTE M_DSI_2 = SUM((M_df1_2 + M_df2_2 + M_df3_2 + M_df4_2 + M_df5_2 + M_df6_2 + M_df7_2 + M_df8_2 + M_df9_2 + M_df10_2 + M_df11_2 + M_df12_2 + M_df13_2 + M_df14_2 + M_df15_2 + M_df16_2 + M_df17_2 + M_df18_2 + M_df19_2 + M_df20_2 + M_df21_2 + M_df22_2 + M_df23_2)/23).

D14. Summed Number of Stressful Life Events in the Last Year [MALE]

Participants chose among the list of 10 stressful events and indicated whether it happened in the last year.

All selected stressful events were summed to create a total score.

*Note*. Due to differences in the two datasets (i.e., Spanish, and Flourishing Families Project ) only stressful life events in the last year were used, as opposed to including those that happened more than a year ago also.

**COMBINED VARIABLES**

D 15. Combined Female and Male Differentiation of Self Inventory: Time 1

COMPUTE DSI_FM_1 = SUM(F_DSI_1 + M_DSI_1).

D 16. Combined Female and Male Differentiation of Self Inventory: Time 2

COMPUTE DSI_FM_2 = SUM(F_DSI_2 + M_DSI_2).
