# Supplementary material for: Differentiation of self and relationship attachment, quality, and stability: A path analysis of dyadic and longitudinal data from Spanish and U.S. couples
Source: PLoS One. 2023 Mar 2;18(3):e0282482. doi: 10.1371/journal.pone.0282482 (PMC9980780; doi:10.1371/journal.pone.0282482)
Supplement: S3 File — (DOCX) [file pone.0282482.s004.docx]

ITLE: US & Spain Cross Cultural Shared Reality: Time 1 Combined DoS on Time 2 Outcomes

DATA: File is FFP data_v3.0.dat;

VARIABLE: Names are id Ctry AGEf1 AGEm1

RQf2 ANXf2 AVDf2 STRf2 INSf2

RQm2 ANXm2 AVDm2 STRm2 INSm2

DSIFM1 DSIFM2;

USEVARIABLES are

Ctry AGEf1 AGEm1

RQf2 ANXf2 AVDf2 STRf2 INSf2

RQm2 ANXm2 AVDm2 STRm2 INSm2

DSIFM1 DSIFM2;

Missing is all (-99);

GROUPING is Ctry (0 = USA 1 = SPN);

ANALYSIS:

TYPE = General;

Estimator = ML;

Bootstrap = 10000;

MODEL:

!Female effects

STRf2 on AGEf1;

RQf2 on DSIfm1;

INSf2 on DSIfm1;

ANXf2 on DSIfm1;

AVDf2 on DSIfm1;

STRf2 on DSIfm1;

!Male effects

STRm2 on AGEm1;

RQm2 on DSIfm1;

INSm2 on DSIfm1;

ANXm2 on DSIfm1;

AVDm2 on DSIfm1;

STRm2 on DSIfm1;

!Combined Covariate(s)

DSIfm2 on DSIfm1;

!Covariance of ALL DVs (Actor)

RQf2 with INSf2;

RQf2 with ANXf2;

RQF2 with AVDf2;

RQF2 with STRf2;

INSf2 with ANXf2;

INSf2 with AVDf2;

INSf2 with STRf2;

ANXf2 with AVDf2;

ANXf2 with STRf2;

AVDf2 with STRf2;

RQm2 with INSm2;

RQm2 with ANXm2;

RQm2 with AVDm2;

RQm2 with STRm2;

INSm2 with ANXm2;

INSm2 with AVDm2;

INSm2 with STRm2;

ANXm2 with AVDm2;

ANXm2 with STRm2;

AVDm2 with STRm2;

!Covariance of ALL DVs (Partner)

RQf2 with INSm2;

RQf2 with ANXm2;

RQF2 with AVDm2;

RQF2 with STRm2;

INSf2 with ANXm2;

INSf2 with AVDm2;

INSf2 with STRm2;

ANXf2 with AVDm2;

ANXf2 with STRm2;

AVDf2 with STRm2;

RQf2 with INSm2;

RQf2 with ANXm2;

RQf2 with AVDm2;

RQf2 with STRm2;

INSf2 with ANXm2;

INSf2 with AVDm2;

INSf2 with STRm2;

ANXf2 with AVDm2;

ANXf2 with STRm2;

AVDf2 with STRm2;

MODEL USA:

!Female Effects

[RQf2] (b0g0);

RQf2 on DSIfm1 (b1g0);

[INSf2] (b2g0);

INSf2 on DSIfm1 (b3g0);

[ANXf2] (b4g0);

ANXf2 on DSIfm1 (b5g0);

[AVDf2] (b6g0);

AVDf2 on DSIfm1 (b7g0);

[STRf2];

STRf2 on DSIfm1 (b8g0);

STRf2 on AGEf1 (b10g0);

!Actor Effects (male)

[RQm2] (b16g0);

RQm2 on DSIfm1 (b17g0);

[INSm2] (b18g0);

INSm2 on DSIfm1 (b19g0);

[ANXm2] (b20g0);

ANXm2 on DSIfm1 (b21g0);

[AVDm2] (b22g0);

AVDm2 on DSIfm1 (b23g0);

[STRm2] (b24g0);

STRm2 on DSIfm1 (b25g0);

STRm2 on AGEm1 (b28g0);

!Combined Covariate(s)

[DSIfm1] (b34g0);

DSIfm2 on DSIfm1 (b9g0);

!Covariance of ALL DVs (Actor)

RQf2 with INSf2;

RQf2 with ANXf2;

RQF2 with AVDf2;

RQF2 with STRf2;

INSf2 with ANXf2;

INSf2 with AVDf2;

INSf2 with STRf2;

ANXf2 with AVDf2;

ANXf2 with STRf2;

AVDf2 with STRf2;

RQm2 with INSm2;

RQm2 with ANXm2;

RQm2 with AVDm2;

RQm2 with STRm2;

INSm2 with ANXm2;

INSm2 with AVDm2;

INSm2 with STRm2;

ANXm2 with AVDm2;

ANXm2 with STRm2;

AVDm2 with STRm2;

!Covariance of ALL DVs (Partner)

RQf2 with INSm2;

RQf2 with ANXm2;

RQF2 with AVDm2;

RQF2 with STRm2;

INSf2 with ANXm2;

INSf2 with AVDm2;

INSf2 with STRm2;

ANXf2 with AVDm2;

ANXf2 with STRm2;

AVDf2 with STRm2;

RQf2 with INSm2;

RQf2 with ANXm2;

RQf2 with AVDm2;

RQf2 with STRm2;

INSf2 with ANXm2;

INSf2 with AVDm2;

INSf2 with STRm2;

ANXf2 with AVDm2;

ANXf2 with STRm2;

AVDf2 with STRm2;

MODEL SPN:

!Female Effects

[RQf2] (b0g1);

RQf2 on DSIfm1 (b1g1);

[INSf2] (b2g1);

INSf2 on DSIfm1 (b3g1);

[ANXf2] (b4g1);

ANXf2 on DSIfm1 (b5g1);

[AVDf2] (b6g1);

AVDf2 on DSIfm1 (b7g1);

[STRf2];

STRf2 on DSIfm1 (b8g1);

STRf2 on AGEf1 (b10g1);

!Actor Effects (male)

[RQm2] (b16g1);

RQm2 on DSIfm1 (b17g1);

[INSm2] (b18g1);

INSm2 on DSIfm1 (b19g1);

[ANXm2] (b20g1);

ANXm2 on DSIfm1 (b21g1);

[AVDm2] (b22g1);

AVDm2 on DSIfm1 (b23g1);

[STRm2] (b24g1);

STRm2 on DSIfm1 (b25g1);

STRm2 on AGEm1 (b28g1); !Control

!Combined covariates

[DSIfm1] (b34g1);

DSIfm2 on DSIfm1 (b9g1); !Control

!Covariance of ALL DVs (Actor)

RQf2 with INSf2;

RQf2 with ANXf2;

RQF2 with AVDf2;

RQF2 with STRf2;

INSf2 with ANXf2;

INSf2 with AVDf2;

INSf2 with STRf2;

ANXf2 with AVDf2;

ANXf2 with STRf2;

AVDf2 with STRf2;

RQm2 with INSm2;

RQm2 with ANXm2;

RQm2 with AVDm2;

RQm2 with STRm2;

INSm2 with ANXm2;

INSm2 with AVDm2;

INSm2 with STRm2;

ANXm2 with AVDm2;

ANXm2 with STRm2;

AVDm2 with STRm2;

!Covariance of ALL DVs (Partner)

RQf2 with INSm2;

RQf2 with ANXm2;

RQF2 with AVDm2;

RQF2 with STRm2;

INSf2 with ANXm2;

INSf2 with AVDm2;

INSf2 with STRm2;

ANXf2 with AVDm2;

ANXf2 with STRm2;

AVDf2 with STRm2;

RQf2 with INSm2;

RQf2 with ANXm2;

RQf2 with AVDm2;

RQf2 with STRm2;

INSf2 with ANXm2;

INSf2 with AVDm2;

INSf2 with STRm2;

ANXf2 with AVDm2;

ANXf2 with STRm2;

AVDf2 with STRm2;

MODEL CONSTRAINT:

NEW

(bRQf bINSf bANXf bAVDf bSTRf bAGEf

bRQm bINSm bANXm bAVDm bSTRm bAGEm bDSIfm);

bRQf = b1g1 - b1g0;

bINSf = b3g1 - b3g0;

bANXf = b5g1 - b5g0;

bAVDf = b7g1 - b7g0;

bSTRf = b8g1 - b8g0;

bAGEf = b10g1 - b10g0;

bRQm = b17g1 - b17g0;

bINSm = b19g1 - b19g0;

bANXm = b21g1 - b21g0;

bAVDm = b23g1 - b23g0;

bSTRm = b25g1 - b25g0;

bAGEm = b28g1 - b28g0;

bDSIfm= b9g1 - b9g0;

PLOT(RQf2USA RQf2SPN);

LOOP(DSIf1, 1,6,1.0);

RQf2USA = b0g0 + b2g0*DSIf1;

RQf2SPN = b0g1 + b2g1*DSIf1;

PLOT:

Type is plot2;

OUTPUT:

STAND CINT(bcbootstrap);
